# Affective and Engagement Issues in the Conception and Assessment of a Robot-Assisted Psychomotor Therapy for Persons with Dementia

**DOI:** 10.3389/fpsyg.2017.00950

**Published:** 2017-06-30

**Authors:** Natacha Rouaix, Laure Retru-Chavastel, Anne-Sophie Rigaud, Clotilde Monnet, Hermine Lenoir, Maribel Pino

**Affiliations:** ^1^Sciences and Technology, Université Pierre et Marie CurieParis, France; ^2^Arts et Métiers ParisTechParis, France; ^3^Broca Hospital, Assistance Publique-Hôpitaux de ParisParis, France; ^4^LUSAGE Living Lab, Research Unit EA4468, Faculty of Medicine, Paris Descartes UniversityParis, France; ^5^CEN STIMCOParis, France

**Keywords:** dementia, social robots, engagement, geriatrics, psychomotor therapy, control software

## Abstract

The interest in robot-assisted therapies (RAT) for dementia care has grown steadily in recent years. However, RAT using humanoid robots is still a novel practice for which the adhesion mechanisms, indications and benefits remain unclear. Also, little is known about how the robot's behavioral and affective style might promote engagement of persons with dementia (PwD) in RAT. The present study sought to investigate the use of a humanoid robot in a psychomotor therapy for PwD. We examined the robot's potential to engage participants in the intervention and its effect on their emotional state. A brief psychomotor therapy program involving the robot as the therapist's assistant was created. For this purpose, a corpus of social and physical behaviors for the robot and a “control software” for customizing the program and operating the robot were also designed. Particular attention was given to components of the RAT that could promote participant's engagement (robot's interaction style, personalization of contents). In the pilot assessment of the intervention nine PwD (7 women and 2 men, *M* age = 86 y/o) hospitalized in a geriatrics unit participated in four individual therapy sessions: one classic therapy (CT) session (patient- therapist) and three RAT sessions (patient-therapist-robot). Outcome criteria for the evaluation of the intervention included: participant's engagement, emotional state and well-being; satisfaction of the intervention, appreciation of the robot, and empathy-related behaviors in human-robot interaction (HRI). Results showed a high constructive engagement in both CT and RAT sessions. More positive emotional responses in participants were observed in RAT compared to CT. RAT sessions were better appreciated than CT sessions. The use of a social robot as a mediating tool appeared to promote the involvement of PwD in the therapeutic intervention increasing their immediate wellbeing and satisfaction.

## Introduction

**Figure 1:**
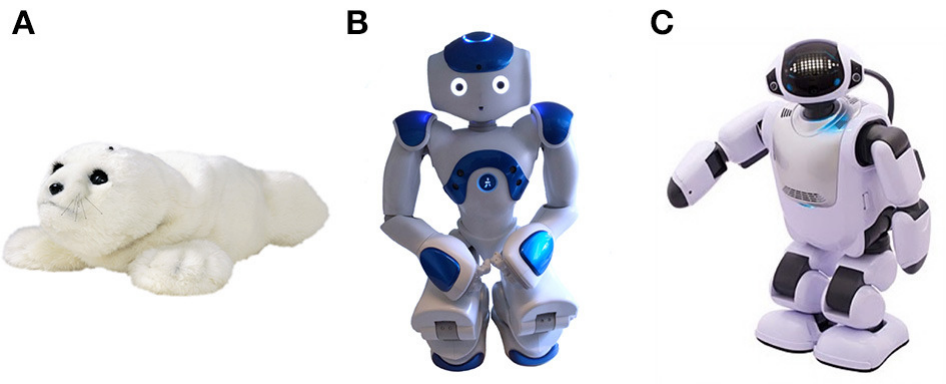
Examples of social robots. **(A)** PARO (AIST, Japan); **(B)** NAO (Softbank robotics, Japan); and **(C)** PALRO (Fujisoft, Japan).

Psychosocial interventions, such as cognitive stimulation, physical activities and art-mediated therapies, play a key role in dementia care. Several studies show a positive impact of these interventions on the well-being, cognition, social life and daily functioning of persons with dementia (PwD) (Hulme et al., 2010; Vernooij-Dassen et al., 2010; Dickson et al., 2012; Oyebode and Parveen, 2016). In recent years a growing number of studies have focused on the use of social robots in interventions for PwD. Social robots offer the possibility of engaging and stimulating the user through social interaction (speech, gestures, behavior). A wide range of robots interpreted as communicative and socially aware fall under this category (Ess et al., 2014), including humanoid, animal-like and some machine-like robots (Figure 1). Most social robots offer a great flexibility of programming allowing the creation of diverse behaviors and customization. For this reason, they have a great potential to support care interventions taking into account inter-individual differences, a well-known success factor in dementia care.

A good number of robot-assisted therapies (RAT) for PwD have used the seal robot PARO (AIST, Japan). Several studies have reported beneficial effects of PARO in PwD, such as an improvement on general well-being and social interaction (Wada and Shibata, 2007), a reduction of stress (Broekens et al., 2009; Mordoch et al., 2013), and diminished use of psychoactive and pain medications (Petersen et al., 2017). Fewer studies have explored the effects of RAT using humanoid robots with elderly persons with cognitive impairment.

López Recio et al. (2013) evaluated the feasibility of using the NAO robot (Softbank robotics, Japan) as an assistant in an individual physiotherapy program with 13 older adults in an assisted living facility. Three conditions were compared: (a) “classic therapy” in which the physiotherapist worked alone, (b) “ViNAO therapy” in which the therapist used a virtual NAO, displayed on a screen, to show the movements the inpatients should mimic and to provide them with feedback; and (c) “PhyNAO therapy” in which the therapist used a real NAO robot for the same purpose. Based on the requirements of the therapist some software modules and a user interface were developed to program NAO's movements and operate it during sessions. A good acceptance by participants was observed. Participants tried to synchronize their movements with those of the robot indicating a good compliance with RAT. One of the advantages of using the robot as an external model was that it allowed the therapist to be more available to mobilize directly the patient. Therefore, the robot contributed to reduce the therapist's workload and improve his interactions with the patients. All participants agreed that the robot's movements were natural and preferred unanimously the real robot to the virtual one. However, it was noted that technical limitations of the robot's hardware affected sometimes the way it performed the exercises (e.g., movements with less amplitude), an inaccuracy that was also mimicked by participants.

Martín et al. (2013) and Valentí Soler et al. (2015) evaluated the use of the NAO robot in cognitive and occupational therapy with 50 elderly PwD in two settings, a day care center and an assisted living facility. NAO was used in individual and group therapy sessions to assist the therapist by playing audio contents and carrying small objects used for the activities. Specific robot's scripts developed for the activity included speech, music and movement. A mobile device was used as remote control by the therapist to operate the robot. Main results from this 3-month experience were a good acceptance of the robot and the improvement of neuropsychiatric symptoms of dementia, such as apathy and irritability, in the group who benefited from the RAT with the NAO.

Results from previously cited studies show that humanoid robots have the potential to provide assistance for psychosocial interventions in dementia care, particularly, when the robot's role and behavior has been defined according to the needs of care professionals and PwD. However, further work is needed to identify the elements of RAT using humanoid robots that are likely to result in clinical improvements in PwD. Moreover, published studies have not dealt in detail with the quality of human-robot interaction (HRI) between PwD and humanoid robots.

In this respect, the assessment of participant's *engagement* in RAT could prove useful. Indeed, one of the factors contributing to the effectiveness of dementia care interventions is their ability to engage participants and ensure their adherence. Engagement in this context has been defined by the act of being occupied or involved with an external stimulus (Cohen-Mansfield et al., 2009). Factors such as the person's characteristics and his/her personal history, the type of stimulus and the environmental conditions in which the activity takes place, all have been found to influence the engagement that a specific individual may have with an activity (Cohen-Mansfield et al., 2010). In recent years, some models for studying engagement of PwD when participating in an activity have been developed and applied to different interventions, for instance the *Observational Assessment of Engagement* (OME) (Cohen-Mansfield et al., 2009) and the *Menorah Park Scale* (Judge et al., 2000). More recently, Jones et al. (2015) developed the *Video Coding Protocol- Incorporating Observed Emotion* (VC-IOE), a specific approach, particularly useful for RAT, to assess engagement in PwD using video coding.

Another aspect that has been little discussed is how to program a humanoid robot to provide PwD with a natural and positive interaction, and consequently, to improve the acceptance of the robot. The work by Hamada et al. (2016) provides some elements in this respect. In their research, they used the social robot PALRO (Fujisoft, Japan) as an assistant in a physical activity therapy for PwD. The robot was used to provide the instructions on how to perform the exercises and to model the movements for the person to follow. The assessment of clinical effects of the intervention was not an objective of this study. Nevertheless, better engagement and satisfaction of participants were reported when the robot's dialogues were accompanied by gestures, when it repeated instructions to enable user's comprehension and when it verbally encouraged and complimented participants. The robot exhibiting a kindly and compassionate attitude proved advantageous in this context.

In their analysis of main challenges of socially assistive robotics, Tapus et al. (2007) explained how giving an empathetic attitude to an assistive robot would benefit HRI. Considering that empathy, the capacity of understanding other's emotions and perspectives, is as a key factor for successful therapeutic relationships, it has been recommended that RAT integrates this aspect. Tisseron et al. (2015) have also suggested that the acceptance of social robots depends on their empathic qualities. These authors proposed a model of empathy extended to four dimensions (i.e., auto-empathy, direct empathy, reciprocal empathy, and intersubjective empathy) and to four components (action, emotion, thought, and assistance) aiming at better understanding HRI.

The main objective of the present study is to investigate the feasibility of using a humanoid robot as an assistant in psychomotor therapy for PwD. The robot's potential to incite the engagement of PwD in the activity and its effect on their emotional state will also be studied. In order to increase RAT acceptance, particular attention will be given to the definition of some components of the RAT: defining a highly acceptable and empathic interaction style for the robot, tailoring the program contents to the preferences and capacities of participants, and creating a framework for RAT based on the triad composed by the therapist, the patient and the robot.

This paper is structured as follows; first we describe the design process of the robot-mediated psychomotor therapy program, including general technical aspects of contents creation and robot programming. Then, we present the experimental pilot study conducted to assess feasibility and immediate effects of the intervention. The last section of the paper provides a general discussion of results and some suggestions for future studies in RAT for dementia care.

## Conception and development of the RAT

### The psychomotor therapy program

A psychomotor therapist conceived a short therapeutic program for PwD structured in four individual sessions: a classic therapy (CT) session, in which the patient was alone with the therapist, and three RAT sessions, in which the therapist was assisted by the robot NAO. Each session comprised five sections described as follows:

1. *Introduction:* Time for greetings and introduction of the robot (RAT).
2. *Motor section:* The section begins with a warm-up exercise by which the person is brought to rediscover and move different parts of his/her body (e.g., head, hands, arms, legs). This exercise should contribute to raise patient's alertness and allows him/her to be physically and mentally available for the session. Then, a sequence of gestural movements is modeled by the therapist (CT), or the robot (RAT), to be repeated step by step and learnt. By stimulating the patient's motor capacities, the therapist also seeks to improve his/her awareness of preserved functional and interaction abilities.
3. *Cognitive stimulation section:* The section begins with some personalized questions tailored to the patient's life history and interests being formulated by the therapist (CT) or the robot (RAT). The second part of this session is devoted to ask the patient some questions about his own body. The purpose of this activity is to elicit verbal exchanges in fields that were familiar to and enjoyed by the patient and to help him/her increase his/her body awareness.
4. *Body expression section*: The patient is invited to imitate a choreography in three steps, associating a sequence of movements to a series of brief meaningless sounds such as “BA, DA, KA.” The sequence is presented and modeled by the therapist (CT) or the robot (RAT). The aim of this section is to stimulate body expression through movement, voice and emotion.
5. *Conclusion:* The session ends with a series of breathing exercises allowing the participant to relax. The exercises are presented and modeled by the therapist (CT) or the robot (RAT). A time of verbal exchange is proposed to the patient at the end of the session.

**Figure 2:**
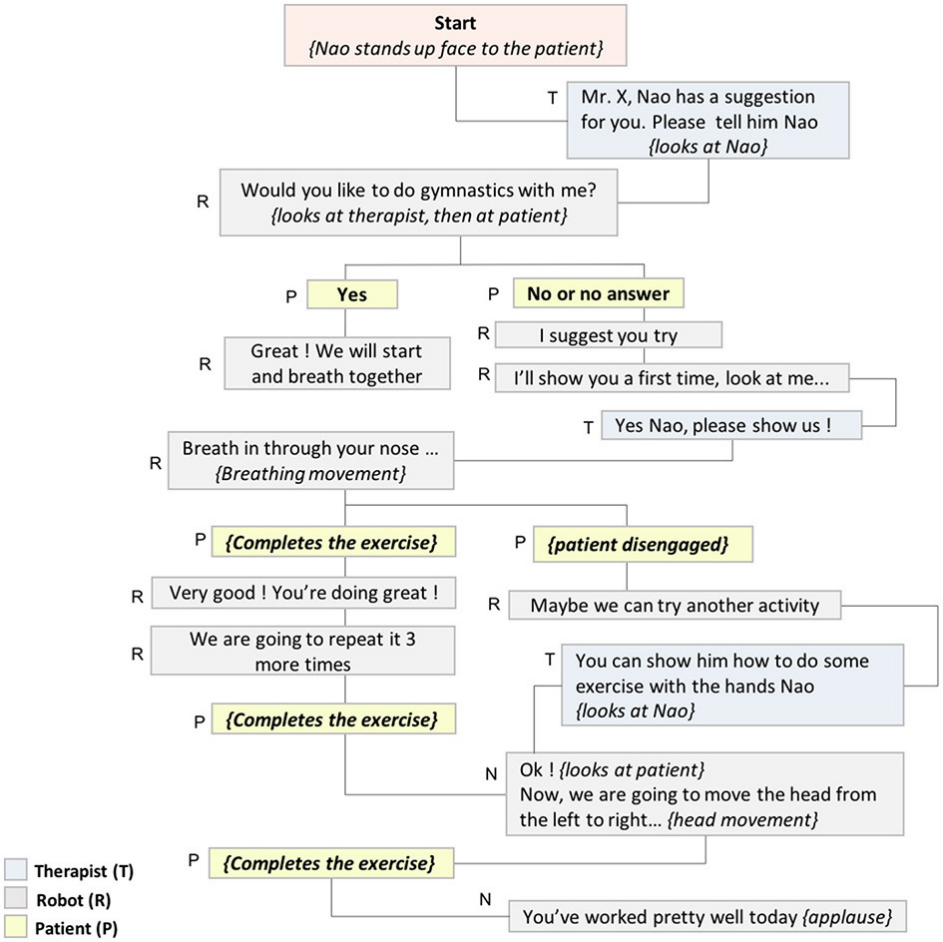
Example of RAT interactive scenario.

Different scenarios were created in order to anticipate possible interaction sequences involving the patient, the therapist and the robot. Verbal and non-verbal robot behaviors required for each sequence were carefully defined taking into account the technical possibilities of the robot (Figure 2). During this process were also identified the “personalization parameters” needed to adapt the program contents to the specific requirements of each participant.

Once the therapeutic program was defined it was submitted for validation by a multi-disciplinary team (two geriatricians, a neuropsychologist and a cognitive psychologist). Then, a computer engineer proceeded to program the robot including its behaviors and personality features. A “control software” allowing the personalization of the therapy sessions and the operation of the robot was also created. During this conception and development phase of the program, the psychomotor therapist and the engineer worked together enabling continuous feedback on the quality of the robot's movements and interactions.

### Robot programming

#### Presentation of the control software

The design of the control software for operating the robot took into account two main criteria: *customization* and *intuitiveness*. Regarding *customization*, the software was designed to adapt the contents of the therapy program and some robot's features to each participant's capabilities and preferences. Customization is a key aspect in dementia care interventions to foster engagement and positive emotional responses. With this purpose, the following customization parameters were implemented: (a) adding the person's name so the robot could use it to address each person in an individualized manner, (b) selecting individual and familiar contents for the therapy activities (music, cognitive stimulation themes, adapted physical exercises,…); (c) adjusting some general robot parameters (e.g., rhythm, voice pitch, volume,…) according to each person's preferences and needs to provide the best possible user experience. *Intuitiveness* of the control interface was highly desired to ensure an easy navigation during therapy sessions, and so to allow the operator to smoothly initiate and stop robot's behaviors. The control software was created using Python language and the user interface was created with the program Qt Designer.

**Figure 3:**
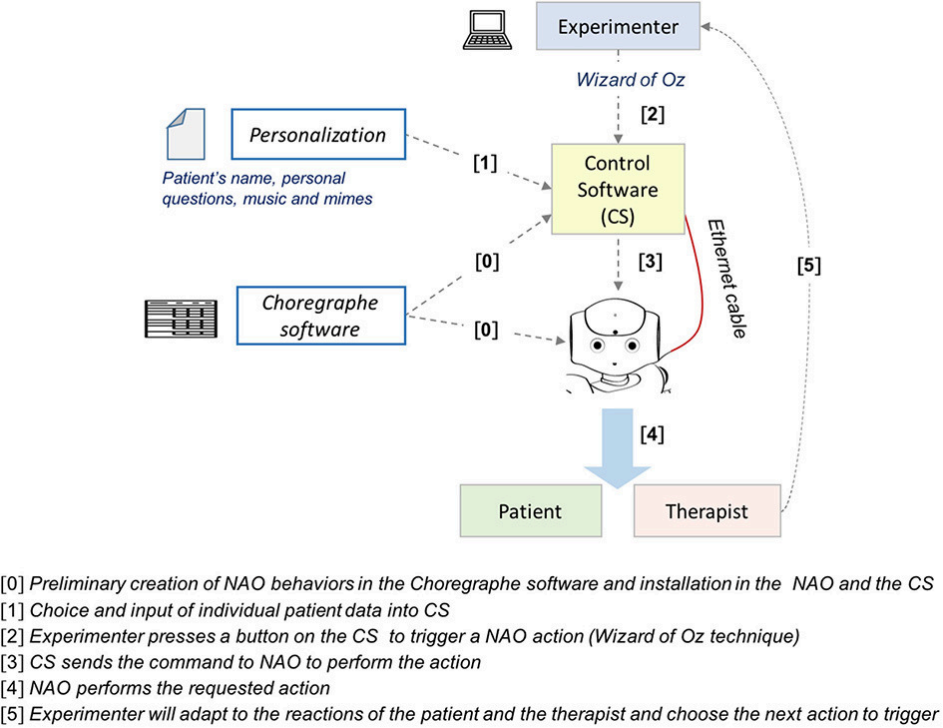
Diagram of the system and principles of operation.

The control software encompassed two kind of files: structure and design files. The *structure files* which contained the raw code to run the software were: (a) the core module, and (b) the associated modules, used to define the functionalities related to movements, audio contents, properties, and software buttons. *Design files* contained the code to set and view the user interfaces. The connection to a virtual NAO robot (Choregraphe software) was set up in order to facilitate the implementation and testing of the robot's behaviors without having to connect the robot in real-time. Figure 3 shows a schematic diagram of the system and the principles of its operation within the context of the present study.

##### Main control interface

**Figure 4:**
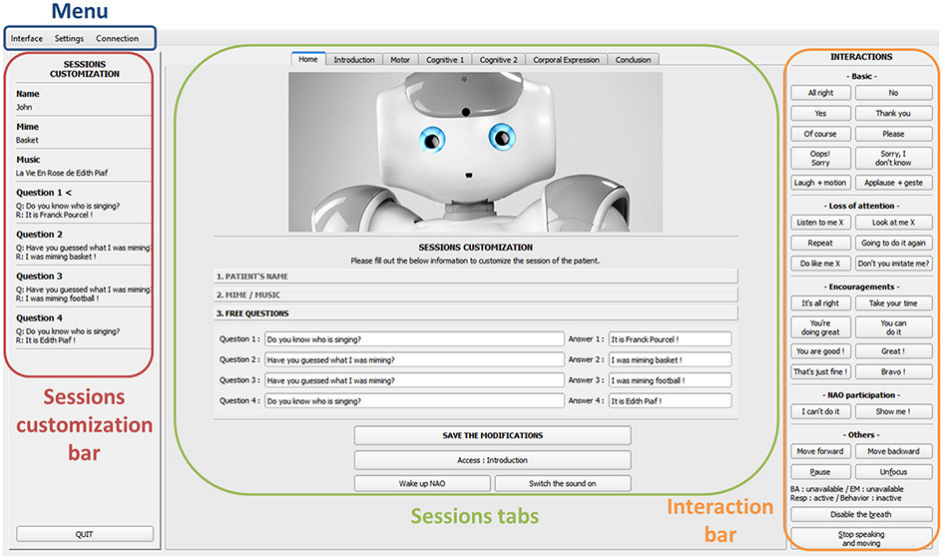
Screen capture of the main control interface.

The main control interface's *central menu* (green box in Figure 4) included seven tabs controls: one tab to personalize the session and six tabs to manage each session section. Functionalities handled by each control tab are described in Table 1.

**Table 1:** Description of control tabs from the main interface.

**Control tab**	**Description**
Home	Set of parameters allowing personalization of the session : *participant's name, personalized content for music themes, themes for cognitive stimulation (questions/answers), and mimes for the session*.
Introduction	Set of parameters allowing the robot to greet participants, introduce itself and make a first “well-mannered” contact with the user: *asking the participant how he feels, or what he did for a living; the robot can laugh if the user touches its head*.
Motor	Set of parameters used by the robot to introduce and model the physical exercises: *the robot explains and performs breathing exercises (inhale and exhale), warm-up exercises, and various sequences of movements*.
Cognitive stimulation 1	Set of parameters used by the robot to introduce and formulate cognitive exercises: *playing music themes, performing a mime, asking questions, giving the answer to a question when the participant is not able to answer*.
Cognitive stimulation 2	Set of parameters used by the robot to ask user questions about his/her body knowledge according to his/her level of cognitive impairment (three levels of difficulty) and to provide guidance in case of error: “*touch my head,” “touch my right shoulder with your right index finger”; “I think this is my left shoulder,” or robot showing the answer using its body*.
Body expression	Set of parameters used by the robot to explain and perform a sequence of movements associated with sounds.
Conclusion	Set of parameters used by the robot to thank the user for participating in the activity, say “*goodbye”* with a yawn, bending and switching off.

The control interface, at the top of the screen, included a menu (blue box in Figure 4) with three options: (a) “Interface,” (b) “Settings,” for customizing the robot's parameters and the session contents, and (c) “Connection,” for connecting the robot. On the left (red box in Figure 4) a “Session customization bar” contained pre-programmed information recorded for a particular session for each individual participant. On the right (orange box in Figure 4) an “Interaction bar” allowing the operator to make the robot quickly react to various user's requests or responses. Options from this interaction bar allowed to make HRI smooth, for instance giving continuity to the conversations between the robot and the therapist or the participant, using basic transition words and accompanying gestures (e.g., “*All right,” “Sorry, I didn't know,” “Laugh* + *motion,” “Applause* + *gesture”)*.

Additional options were proposed to deal with the loss of attention of the user (e.g., “*Don't you imitate me?” “Listen to me X (name of the person),” “Look at me X (name of the person)”*), to regularly encourage and praise the user (e.g., “*You're doing great,” “Take your time,” “You can do it”*), to react when the user requested the robot to make something the robot wasn't programmed for (e.g., “*I can't do it,” “Show me”*), and finally to operate other robot's behaviors (e.g., walking toward and backward, making a pause, stop speaking and moving).

**Figure 5:**
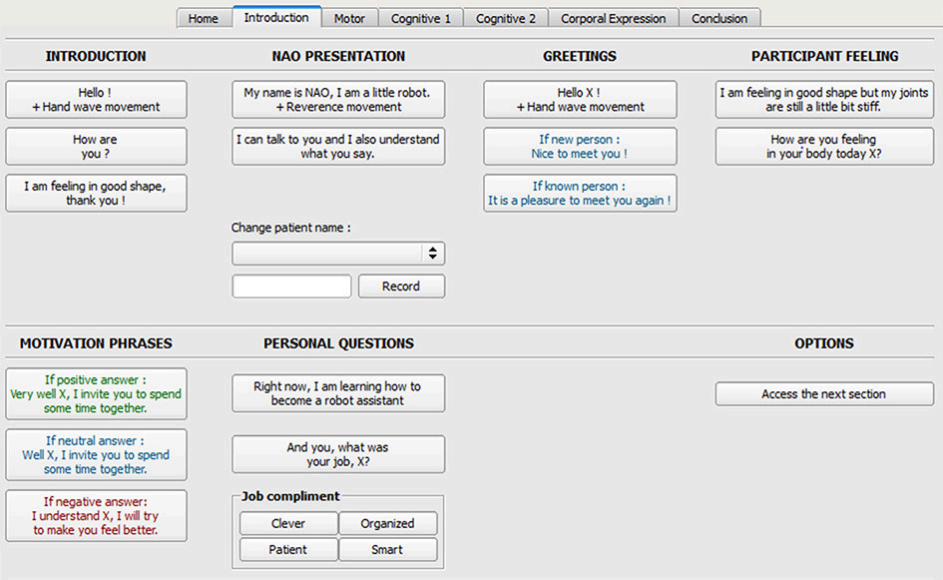
Detail of the control tab for the “Introduction” section.

Each tab of the main interface (Figure 5) corresponding to different parts of the session included a set of buttons sorted by categories allowing a flexible leading of the session according to the participant's responses. Robot's actions were summarized on each button of the interface following a logic “dialogues to say” and “movements to achieve.” For example, “*Hello! X* + *Hand wave”* means that the robot says “*Hello! X”* and waves its hand to say hello (where X is the name of the patient). See Supplementary Material for the presentation of control interfaces for each subsection of the program.

##### Secondary interfaces

**Figure 6:**
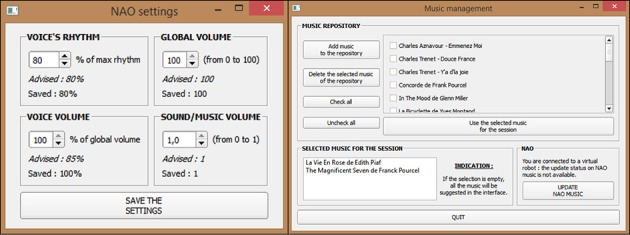
Music settings manager and Audio settings manager.

Three managers were accessible from the “Settings” menu on the main control interface to handle mime exercises, music and audio settings in an easy way (Figure 6). For example, the music settings manager allowed adding and deleting music themes to the music folder of the software and selecting the musical themes for the session according to each participant's preferences, without using the Choregraphe software. The mime exercises manager worked in a similar way but it required having created an associated behavior via Choregraphe beforehand. The audio settings manager allowed the modification in real time of volume and voice parameters of the robot. Personalized parameters, once registered, were held in the software memory and displayed when reopening each individual session.

#### Personality features of the robot

Effort was put on giving the robot an empathic and a positive attitude (e.g., being warm, polite, supportive, tolerant, gracious…). Some empathy signs, such as (a) the ability to recognize other person's emotions; (b) to communicate with persons; (c) to display emotions; and (d) to take perspective (Tapus et al., 2007), were considered when defining the robot's behavior and personality. Three other principles proposed in the field of HRI were also used in this process: (a) *interactivity*, the robot coexists with an interactive person in the same time-space continuum; (b) *equifinality*, the robot is able to adapt to each person and the same objective may be reached in different ways; and (c) *multimodality*, the robot is able to interact with a human using different communication channels (e.g., verbal, tactile, kinesthetic, or emotional) (Libin and Libin, 2004). Table 2 presents a summary of robot's behaviors and personality traits related to the aforementioned dimensions that were implemented in this work.

**Table 2:** Robot's behaviors related to different HRI dimensions.

**Dimension**	**Behaviors, attitudes, personality traits**
Empathy	Displays an emotional state and is able to acknowledge the participant's emotions and feelings. Programmed to exhibit empathic gestures such as giving confirmation signs by head movements. Expresses its own opinions. Gives positive feedback and frequently acknowledges the participant's performance, boosting his/her confidence and motivation.
Interactivity	Robot's embodiment is exploited in order to inspire participants the attribution of intentions, goals, and a personality to the robot. The robot, often compared to a child for its size and appearance, is designed to answer and behave like a “well-mannered” child using simple sentences and childlike gestures. The robot is programmed to automatically move its upper limbs when speaking to support verbal communication through body language. When the robot is not talking, it is programmed to slightly undulate, giving the impression of breathing and being alive. Regarding *proxemics*, the robot is placed on the ground so that the user has a higher view on it and dominates it. The robot is placed at a distance of about 1.50 m from the person which represents the social distance of interactions with friends and colleagues (Hall, 1966). This distance can be adjusted during the interaction to fit the dynamic of the session. Before walking, the robot warns the person communicating the adjustment of the interactive distance.
Multimodality	The robot shows engagement to its interlocutor through gaze and speech (e.g., “*do as I do X*” or “*look at me X”*). If the participant interrupts it, the robot is programmed to stop talking or making a movement and return to its initial position. Robot's speech and gaze are programmed to face directly its interlocutor using the Face Detection application. •When the user touches the robot, it is programmed to laugh. At the end of the session, it is programmed to stretch and yawn before switching off.
Equifinality	Before each session, the robot's behavior and RAT contents were customized for each user. A set of basic and transition answers like “*yes,” “no,” “thank you,” “please,” “I don't know,”* were implemented to ensure the robot provides appropriate responses to each participant's requests. The communication style of the robot was tailored to the abilities of older adults with cognitive disorders (e.g., simple vocabulary, short sentences). When the robot's comments are not understood by the participant the robots is programmed to repeat the sentence.

## Materials and methods

### Study design

An exploratory study aiming to assess the feasibility and immediate effects of a psychomotor therapy program for PwD using the NAO robot as an assistant was conducted between February and May 2016 in the Broca Geriatric Hospital (Paris). The intervention program consisted in 4 individual sessions of psychomotor therapy including: one classical psychomotor therapy session (CT) (therapist-patient) and 3 RAT sessions (therapist-patient-robot).

### Participants

Nine persons (7 women and 2 men, mean age 86 years) hospitalized in a geriatrics unit, took part in the study. Inclusion criteria were: having a clinical diagnosis of neurodegenerative dementia and having signed a consent form. Exclusion criteria were: severe dementia (MMSE <10/30), sensory deficit (vision and hearing) and severe acute illness impeding the participation in RAT sessions.

### Tools

- A NAO robot, Version V4 (Softbank robotics).
- The “Choregraphe” software (Softbank robotics), a multi-platform application allowing the creation of behaviors for the NAO robot, its monitoring and control (version 2.1).
- A “home-made” software developed to create robot's behaviors, customize sessions, and monitoring and control the robot. The software is described in Section The Psychomotor Therapy Program.
- “The Observer XT” software, version 11.5 (Noldus), for video-based behavioral analysis.

#### Psychosocial assessment tools

- The “Mini Mental State Examination” (MMSE) (Folstein et al., 1975), for general cognitive assessment. Scores range from 0 (major cognitive impairment) to 30 (normal cognitive functioning).
- The “Neuropsychiatric Inventory-Nursing team version” (NPI-ES) (Sisco et al., 2000) for the assessment of behavioral symptoms in PwD by the nursing staff. NPI comprises 10 dimensions: delusions, hallucinations, dysphoria, apathy, euphoria, disinhibition, aggressiveness and agitation, irritability, anxiety, aberrant motor activity. Scores range from 0 to 120. Highest scores correspond to major behavioral disturbances.
- The “Self-Identity Questionnaire” (SQI) (Judge et al., 2000), used to establish a profile of customized activities for PwD, taking into account their interests and preferences.
- The “International Positive and Negative Affect Schedule Short-Form” (I-PANAS-SF) (Karim et al., 2011), used to quantify a person's emotional state in the short term, with 10 items representing either positive or negative affects (two scores ranging from 0 to 25).
- The “Instant Assessment of Wellbeing Tool” (EVIBE), for assessing immediate wellbeing and quality of life of elderly people in nursing homes (Kuhnel et al., 2014). Scores range from 1 (sadness) to 5 (happiness).
- The “Menorah Park Engagement Scale” (MPES) (Judge et al., 2000), for measuring the amount and types of engagement by PwD in the course of an activity based on behavioral analyses. Two adaptations were made to the MPES for the present study: (a) a “*robot engagement”* category was created to specify participant's emotional and behavioral responses denoting an exclusive engagement toward the robot (i.e., unrelated to the target activity), (b) an “*at ease/relaxed”* category was added to the emotional engagement dimension in order to take into account the flat affect and limited facial emotion responses commonly observed in PwD. Table 3 presents a summary of the MEPS engagement categories and examples of responses within the context of this study.

**Table 3:** Summary of the Menorah Park Engagement Scale (MEPS) dimensions and examples of coding.

**Type of engagement**	**Definition**	**Example of response coded**
**BEHAVIORAL DIMENSION**		
Constructive Engagement (CE)	The person participates in the target activity. This includes motor and verbal responses in response to the target activity (e.g., commenting or making a gesture/action)	Participant responds to the therapist questions or instructions either verbally or by executing the physical movement required
Passive Engagement (PE)	The person listens to or looks at the target activity without making the actions required by the activity (repeating a movement/gesture or answering a question)	Participant watches the physical movement exercise presented by the therapist but does not reproduce the movement at his/her turn
Other Engagement (OE)	The person pays attention to something other than the target activity or does something not related to the target activity (speaking, gesturing, watching or listening to)	Participant looks out the window and talks about what he/she sees
Engagement with the robot not related to the target activity (RE)	The person is disengaged from the target activity and focuses his/her attention on the robot (touches the robot, speaks to the robot…)	Participant disengages from the therapy to interact verbally or physically with the robot in a way not related to the target activity: *"NAO, do you have a girlfriend?*
Non-engagement (NE)	The person does not participate in the target activity in any way	Participant sleeps, closes his/her eyes or stares into space
**Emotion**	**Definition**	**Example of coding**
**EMOTIONAL DIMENSION**		
Pleasure	The person clearly laughs, smiles or verbalizes a positive response/emotion during the activity	Participant distinctly shows and/or verbalizes a positive emotion: *"I'm happy," “It makes me feel good”*
Anxiety/sadness	The person cries, looks sad, looks down, shows a tight facial expression, or verbalizes a negative response/emotion during the activity	Participant shows and/or verbalizes a negative emotion “*I feel useless,” “it makes me feel sad”*
At ease/relaxed	The person is calmed, peaceful, comfortable at the activity	Person appears serene, shows a neutral expression

Additionally, two Visual Analogic Scale (VAS) were built for the purposes of this study. One to assess the satisfaction of participants regarding each therapy session (Question was: *Did you enjoy the session?*); and the other to evaluate the pleasure while using the robot in RAT sessions (Question was: *Did you enjoy the presence of the robot?)* Each VAS was scored between 1 and 5 (highest values translated most positive opinions).

### Procedure

The protocol of the study was explained to the Geriatrics Unit nursing staff and the geriatrician (MD) responsible for the unit who helped to identify the patients who met the criteria to take part in the trial. Two researchers contacted each potential participant and his/her relatives and gave them details on the study and the intervention. If the patient had given verbal consent to participate, an appointment was scheduled in order to make the inclusion. This study was carried out in accordance with the recommendations of Paris Descartes ethical procedures and included written informed consent from all subjects according to the Declaration of Helsinki.

**Figure 7:**
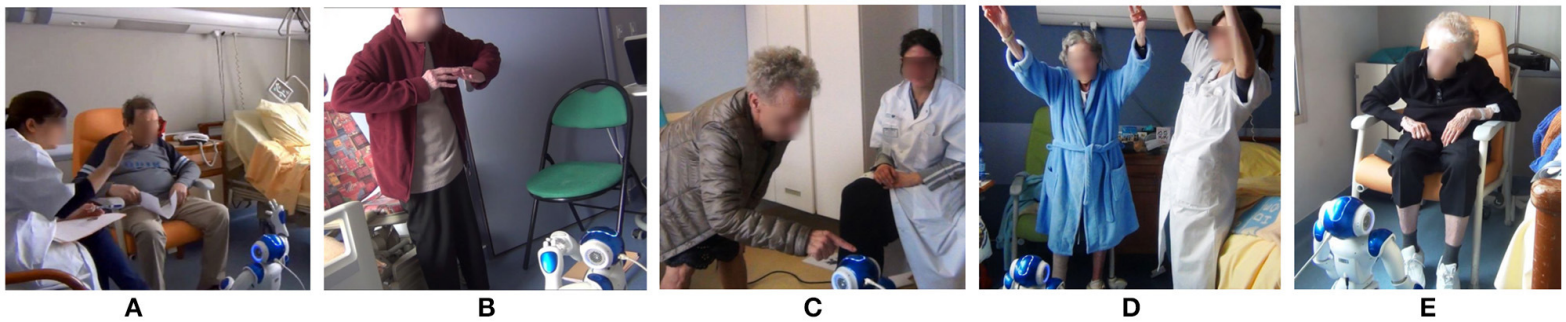
Robot-assisted psychomotor therapy sessions. **(A)** Introduction, **(B)** motor section **(C)** cognitive Stimulation, **(D)** body expression section and **(E)** conclusion.

On the day of the inclusion, after written consent was obtained, a clinician collected socio-demographic data and conducted the baseline neuropsychological assessment for the definition of the participant's profile (see Table 4). The experimental protocol consisted of four individual non-consecutive sessions over a period of 5 weeks: one CT session and three RAT sessions. Figure 7 illustrates the different moments of the RAT sessions. Outcome variables were measured throughout the experimentation according to the schedule shown in Table 5.

**Table 4:** Demographic and clinical characteristics of the sample.

**N°**	**Gender**	**Age**	**Education level**	**Diagnostic**	**MMSE (0–30)**	**NPI –ES dominant profile**	**NPI-ES (0–120)**
1	Female	68	6	Alzheimer's disease	15	Agitation	5
2	Female	88	6	Parkinson's disease	22	Anxiety	15
3	Female	90	4	Mixed dementia	16	Agitation	12
4	Female	95	3	Mixed dementia	12	Dysphoria/depression	7
5	Female	92	7	Alzheimer's disease	16	Apathy	15
6	Male	92	7	Lewy body dementia	12	Agitation	12
7	Male	84	7	Mixed dementia	13	Apathy	14
8	Female	89	4	Neurodegenerative disease	19	Anxiety	7
9	Female	76	4	Neurodegenerative disease	19	Apathy	3

*EL, Education level, ranging from 1 (validation of primary school) to 7 (higher education degree); MMSE, Mini Mental State Examination; NPI-ES, Neuropsychiatric Inventory-Nursing team version*.

**Table 5:** Evaluation criteria and schedule of assessments throughout the experimentation.

**Assessment criteria**	**Tool**	**Baseline**	**CT**	**RAT1**	**RAT2**	**RAT3**	**Post**
Cognitive functioning	MMSE	✓	-	-	-	-	-
Neuropsychiatric symptoms	NPI	✓	-	-	-	-	-
Life history and preferences	SQI	✓	-	-	-	-	-
Emotional state	PANAS	✓					✓
Immediate wellbeing	EVIBE	-	*Pre Post*	*Pre Post*	*Pre Post*	*Pre Post*	-
Engagement	MPES	-	✓	✓	✓	✓	-
Satisfaction with intervention	VAS	-	✓	✓	✓	✓	-
Appreciation of robot	VAS	-	-	✓	✓	✓	-
Verbal and nonverbal empathy related behaviors	Video analysis	-	-	✓	✓	✓	-

*CT, Classic Therapy; RAT, Robot-Assisted Therapy (1,2,3 for sessions 1,2,3 respectively); Post, assessment after intervention; MMSE, Mini Mental State Examination; NPI–ES, Neuropsychiatric Inventory-Nursing team version; SQI, Self-Identity Questionnaire; PANAS, International Positive and Negative Affect Schedule; EVIBE, Instant Assessment of Wellbeing Tool; MPES, Menorah Park Engagement Scale; VAS, Visual Analogic Scales; Pre Post, assessment before and at the end of each therapy session, fields marked with a ✓ indicate that the variable was assessed at that time point; fields marked with a — indicate that the variable was not assessed at that time point*.

Therapy sessions were held in the patient's hospital room. The patient was seated on a chair facing the therapist, and the robot in RAT sessions. The experimenter (engineer) who operated the robot was sitting back in the room with the computer which remained visible to the participant. The experimenter used the Wizard of Oz (WOZ) technique to remotely control the robot's movements, speech, and gestures (Kelley, 1984).

### Data analysis

The encoding and analysis of the video recordings was carried out by two researchers using the adapted form of the MPES (Judge et al., 2000). The order of video analysis was randomized. Analysis of the engagement was performed using time percentage with respect of the total time of each session's section (motor, cognitive stimulation, and body expression). Statistical analyses of neuropsychological measures were performed using the Wilcoxon test to compare means. For these analyzes, the significance level used was 95% (alpha = 0.05).

## Results

### General results

A total of 35 therapy sessions were conducted: 8 CT sessions and 27 RAT sessions. The sessions had a mean duration of 22.15 min, for a total of 770.19 min altogether that were video-analyzed. Table 6 presents mean duration of the sessions detailing each subsection. All the participants underwent the four experimental sessions as stated in the protocol, except one participant who refused to take part in the CT session. Table 7 presents a summary of a RAT session.

**Table 6:** Mean duration of the sessions (total and each section's).

**Session**	**Introduction**	**Motor**	**Cognitive stimulation**	**Body expression**	**Conclusion**	**Total**
**MEAN DURATION (MIN)**						
CT	0.57	8.28	7.50	1.55	0.56	18.48
RAT 1	2.70	8.75	9.70	2.50	1.27	25.54
RAT 2	1.34	8.46	9.78	1.93	1.80	23.68
RAT 3	0.19	7.88	8.38	1.67	1.08	20.90
Total mean	1.2	8.34	8.84	1.91	1.18	22.15
SD	1.11	0.36	1.10	0.42	0.51	3.10

**Table 7:** Summary of a RAT session.

**Dialogues**	**Behaviors**
**INTRODUCTION**	
**Therapist:** Hello Mr. X, hello NAO.	*[looks at the patient, then at NAO]*
**NAO:** Hello Mr. X I think that we have already met. I am happy to see you again. *Personalized content: this is the second time Mr. X meets NAO*	*[looks at the patient, waves hand to say hello]*
**Therapist:** How do you feel in your body today NAO?	*[looks at NAO]*
**NAO:** I feel great in my body but my joints are not still well awaken. What about you Mr. X?	*[looks at the patient, head movement]*
**Patient:** I do not feel very well today.	
**NAO:** Ok Mr. X. then I will try to make you feel better with our therapist.	*[arms and head movement]*
**MOTOR SECTION**	
**Therapist:** We will begin by a short awakening, moving the different parts of our body. Which part of your body would you like to move first Mr. X?	*[looks at the patient]*
**Patient:** My hands.	
**Therapist:** NAO, do you have an idea for exercising our hands?	*[looks at NAO]*
**NAO:** Yes of course! We are going to open and close our hands, like this.	*[opens and closes its hands, looks at the patient]*
**NAO:** Now, let's do this together!	*[opens and closes its hands, looks at the patient]*
**Patient:**	*[opens and closes his hands like NAO]*
**NAO:** Very well done Mr. X.	*[affirmative head movement and applause]*
**COGNITIVE STIMULATION SECTION**	
**Therapist:** NAO, now that we have moved pretty well, I suggest that we take some time for speaking together and activating our brain.	*[looks at NAO]*
**Therapist:** Would you like Mr. X, if NAO asks us some riddles?	*[looks at the patient]*
**Patient:** Yes.	
**Therapist:** NAO, could you ask us a riddle about cooking?	*[Looks at NAO]*
**NAO:** Yes, of course! Which ingredients do we need to cook pancakes? *Personalized content: Mr. X. likes cooking*	*[looks at the patient, head and arms movement]*
**Patient:** eggs, flour, sugar, milk and salt!	
**NAO:** Well done! I would love to know as many things as you do once!	*[affirmative head and arms movement]*
**BODY EXPRESSION SECTION**	
**Therapist:** I suggest that we end the session with a shout of joy!	*[looks at the patient and then at NAO]*
**Therapist:** NAO, could you show us a choreography with movements and sounds to set up our shout of joy, please?	*[looks at NAO]*
**NAO:** with pleasure! I am going to show you how to do it for the first time: *“BA DA KA”*	*[NAO speaks loudly and shows the choreography to patient and therapist]*
**NAO:** Now, let's do it together Mr. X.	*[looks at the patient, inviting head and arms movement]*
**Patient:** yes.	*Together patient, therapist and NAO do the choreography and shout “BA DA KA”*
**Therapist:** Now, I suggest to do it again and shout louder!	*[looks at the patient and then at NAO]*
**NAO:** Yes, of course	*Together patient, therapist and NAO do the choreography and shout “BA DA KA” louder than the first time*
**CONCLUSION**	
**Therapist:** Now we have to say goodbye to NAO because it has to rest a little while.	*[looks at the patient, then at NAO]*
**NAO:** I had a very nice time with you Mr X. Goodbye Mr. X.	*[waves hand to say hello]*
**Patient:** Goodbye little boy.	*[looks at NAO]*
**Therapist:** Goodbye NAO.	*[looks at NAO]*
**NAO:**	*[NAO stretches and folds down]*

#### Engagement in the psychomotor intervention

Results indicated a high constructive engagement of participants in both CT and RAT sessions. Table 8 shows the comparison of percentages in time of the different types of engagement for CT and RAT sessions, first for the entire session (all sections included) then for each subsection. To compare the engagement percentages in both conditions (CT and RAT), the values for the three RAT sessions were averaged.

**Table 8:** Mean time percentage for the different types of engagement in CT and RAT sessions.

**Type of engagement MPES**	**Entire session**		**Motor section**		**Cognitive section**		**Body Expression section**	
	**CT**	**RAT**	**CT**	**RAT**	**CT**	**RAT**	**CT**	**RAT**
Constructive engagement	88%	81%	85%	79%	91%	83%	97%	84%
*p*-value	0.069		0.108		0.091		0.176	
Passive engagement	6%	12%	8%	15%	4%	12%	3%	10%
*p*-value	0.069		0.063		0.028^*^		0.138	
Robot engagement	/	5%	/	4%	/	4%	/	5%
Other engagement	5%	2%	7%	2%	4%	1%	0%	1%
*p*-value	0.344		0.075		0.593		0.18	
No engagement	1%	0%	0%	0%	1%	0%	0%	0%

*CT, Classic therapy; RAT, Robot-assisted therapy (mean of the 3 sessions)*;

* *Statistically significant values*.

No significant difference between CT and RAT sessions was observed in any dimension of engagement, except for a significant increase in passive engagement in the Cognitive Stimulation section of RAT sessions. Robot engagement (i.e., participant disengaged from the target activity and focused on the robot) was observed in RAT but its duration was very short to consider the robot as a source of distraction.

We analyzed the relationship between Constructive Engagement, cognitive status (MMSE) and neuropsychiatric symptoms (NPI). The levels of Constructive Engagement in RAT sessions and the severity of neuropsychiatric symptoms were positively correlated (*r* = 0.68, *P* < 0.05, Spearman's rank correlation), showing that patients presenting behavioral symptoms such as apathy or agitation responded well to RAT. The correlation between Constructive Engagement and neuropsychiatric symptoms was not observed for the CT session. Furthermore, no association was observed between cognitive status (MMSE) and Constructive Engagement (independently of the condition).

#### Emotional impact of the intervention

**Figure 8:**
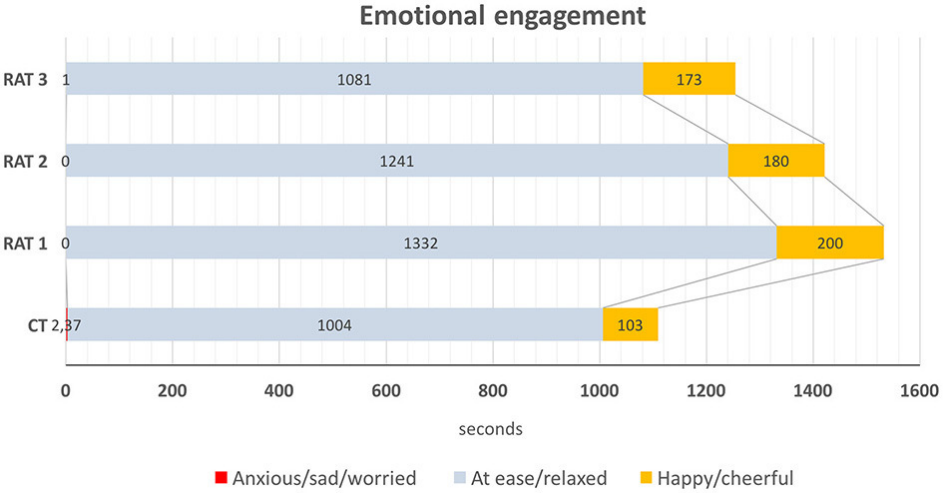
Emotional engagement in CT and RAT sessions.

The emotional impact of the intervention was assessed using the three kinds of responses from the “Emotional engagement” dimension of the MPES: *anxiety/sadness* (tearfulness, depressed affect), *relaxed/at ease* (neutral expression, calmed), and *pleasure-related* (evident manifestations of happiness, cheerfulness). In both conditions participants appeared to be most of the time relaxed and at ease (91% of the time in CT and 87% in RAT). Negative emotional responses were practically non-existent. Obvious pleasure-related responses were noticed during short periods of time, compared to the prevalent neutral/relaxed facial expression of participants during the therapy sessions. Nevertheless, results showed a significant statistical difference (*p* = 0.018) between CT and RAT sessions regarding the duration of pleasure-related responses (9 and 13% respectively) (Figure 8).

Immediate wellbeing (i.e., participant reporting feeling better after the end of the therapeutic session than before) was assessed using the difference in the EVIBE score after and before each therapy session. Highest scores indicate a highest improvement in immediate wellbeing. EVIBE scores showed a greater improvement in wellbeing in RAT sessions than in CT sessions (0.56 vs. 0.22 respectively), but this difference was not statistically significant.

The person's emotional state in the short term was analyzed by comparing the PANAS score at the baseline (baseline) and at the end of the intervention program. Results showed a significant improvement of *positive affects* (e.g., interested, excited, strong, enthusiastic, inspired, proud, alert, determined, attentive, active) (9.78 vs. 13.67, *p* = 0.01) and a decrease of negative affects (distressed, upset, guilty, ashamed, hostile, irritable, nervous, jittery, scared, afraid) (9.56 vs. 7.89, *p* = 1.125) that was not statistically significant.

#### Satisfaction of the intervention and appreciation of the robot

Globally, all participants were satisfied with the intervention program. However, PwD preferred the RAT sessions rather than the CT one (RAT 4.31/5 vs. CT = 3.63/5). This difference regarding the modality of the therapy was statistically significant (*p* = 0.027). The robot was very well accepted by all participants as shown by a satisfaction score of 4.7/5.

#### Empathy related behavior in RAT sessions

During the RAT sessions, various verbal and non-verbal empathy-related behaviors were observed in participants while interacting with the robot. Table 9 provides an overview of the empathy-related behaviors exhibited by the participants. It also includes the number of participants who displayed these behaviors.

**Table 9:** Empathy-related behaviors observed in participants during RAT sessions.

**Type of behavior**	**Examples**	**Number of participants (total *N* = 9)**
Calling the robot by its name	“*Hello NAO”*	7
Giving an affective name (nickname) to the robot or expressing an affective feeling	“*My big one”; “My little one”; “My little chicken”; “I begin to love this little guy”*	4
Speaking directly to the robot without the intervention of the therapist	“*Yes”; “No”; “Thank You”*	8
Using an informal way of addressing the robot	“*You are cool”; “What's up?”*	7
Complementing the robot	“*You are nice”; “You are funny”; “You are cute”; “I like you very much”*	8
Contagious laughter	Smiles and laughs when the robot laughs; “*You make me laugh”*	8
Being receptive to robot's compliments	Smiles or laughs; “*Thank you NAO”; “I am proud of your compliments”*	6
Attributing an emotional state to the robot	Asking the therapists what was the proper way to address the robot using “*Vous”* (formal) or “*Tu”* (familiar); “*Are you tired?”; “Are you happy?”; “Do you like this?”; “Are you laughing at me?”*	8
Attributing an environment or a life history to the robot	Asking whether NAO was a boy or a girl; “*Will you grow up”; “Do you have a girlfriend?”; “Your mother educated you very well”; “What do you eat?”*	4
Attributing the robot the ability to understand one's emotional state	“*I hope that I have not disappointed you”*	2
Positive behavioral manifestations	*Kissing, hugging, touching the robot*	8

Qualitative analysis of video recordings showed that, when talking directly to the robot, three out of nine participants mostly used short sentences (e.g., “*yes”* or “*no”*) and initiated little or no dialogue with it. Among those three PwD, one participant rarely responded to the robot with a nod of his head and mostly answered the question looking at the therapist. The other six participants responded to the robot questions with complex sentences and spontaneously initiated conversations with it. As shown in Table 9, all the adjectives used by the participants to describe the robot were positive.

## Discussion

### Technical aspects

The main advantage derived from the control software created to operate the robot and customize therapy sessions was to conduct the therapeutic sessions in a smooth, fluid and natural way. The WOZ technique, used to tele-operate the robot during the experimentation, enabled the creation of natural, coherent, and timely robot's verbal and non-verbal responses and thus to increase its capacities. However, this choice implied that the robot was not able to perform any automatic behavior. Operating the robot using the WOZ technique required thus a special sensitivity and sustained attention for achieving a high-quality HRI. Besides, the experimenter had to know well how to navigate the control interface and the location and contents of action buttons. In our case it was the developer of the software who played the role of “wizard,” circumstance that simplified the task. However, the use of the control interface by an external user, despite its intuitiveness, would surely require extensive training.

In order to improve the operation of the robot in future work some possibilities can be considered:

- *Automatizing some of the robot's behaviors*, for instance by linking automatically the behaviors of the robot, one after the other, after triggering an action. By implementing this procedure, the number of buttons to handle in the control interface could be reduced and also the number of interventions required from the operator. Still, the risk of “over-automatizing” NAO's behavior is to greatly reduce the naturalness of the interaction.
- *Simplifying the control interface:* this option would require to group by categories different actions of the robot. Following this option, it could be possible to have an initial list of activity sections (e.g., introduction, motor, cognitive, etc.). The operator would then select the category wanted and a menu would display a page grouping again various subcategories of actions according to the choice. Adding a random option for some behaviors, such as the “Encouragements,” that would be operated by using a single button instead of using a specific button for each phrase also goes in this direction;
- *Defining a decisional tree of actions* allowing to link automatically one action with the previous one, as proposed in the study of Sehili et al. (2014). However, although possible, this method would require an important work of reflection and planning to retain the flexibility of the control interface proposed in this study.

Finally, the technical setting used for this study resulted somehow complicated (e.g., transporting and installing the computer, connecting the robot by a cable, needing to accommodate the robot operator in the experimental setting). It would be interesting to adapt the control software to allow its use on a tablet, a smartphone, or any other mobile tool. After simplifying the software, the therapist could be able to operate the robot by himself. This solution has already been put into practice in other studies (Martín et al., 2013).

### Factors of RAT acceptance

Results from this experimental study showed a high level of constructive engagement among PwD throughout the intervention (indistinctly from the condition), increased manifestations of pleasure in RAT sessions, compared to CT sessions, a better appreciation of RAT sessions over CT sessions, and the exhibition of a wide range of empathy-related behaviors of PwD during RAT. All these findings represent good indicators of the advantage of using a humanoid robot for this kind of therapeutic intervention.

The choice of the humanoid robot NAO, the personalization of sessions, the “internal harmony” of the character created, empathy-related responses from the robot, and the characteristics of the therapeutic framework proposed, appeared to have contributed to create a well-accepted RAT intervention. In this section we discuss briefly these aspects:

- The *choice of a humanoid robot*: The humanoid aspect of NAO is a factor that facilitates its acceptance. Previous studies in this area had already confirmed the acceptance of this humanoid robot among elderly users (Wu et al., 2012; López Recio et al., 2013; Martín et al., 2013; Pino et al., 2015; Valentí Soler et al., 2015). Libin and Libin (2004) had also discussed that a key challenge of socially assistive robotics is to create robots that are able to imitate human behavior on the cognitive, motor and emotional level.
- *Personalization:* The flexibility of the NAO programming platform was an asset for the construction of personalized therapeutic sessions. Several studies have shown that dementia care interventions that have the greatest impact on behavioral disorders are those that are adapted to the person's cognitive, motor, and sensory abilities (Cohen-Mansfield et al., 2007) and tailored to the preferences of the person (Gerdner, 2000). The neuropsychological assessment and the use of the self-identity questionnaire (SQI) at the baseline of the experimental study, allowed us to accurately define participants' cognitive profile, and to identify their preferences and interests. This piece of information, used to program the content of the sessions, appeared to support RAT acceptance.
- The “*internal harmony” of the robot:* Another factor that could have contributed to RAT acceptance was the interaction style given to the NAO in our study. Regarding verbal and non-verbal communication, the robot was programmed to use simple sentences for facilitating understanding by elderly persons with cognitive impairment. Some of its behaviors were modeled also to be childlike and non-judgmental, in order to make the robot more likeable. This interactional style used to program robot's behavior was coherent with “childish” aspect of NAO. The concept of “internal coherence,” suggested by Tisseron (2015), could explain the effects of our design choices on robot's acceptance. For this author, the acceptance of a social robot would strongly depend, not on its aspect but on its “internal harmony.” This means, the coherence between its appearance and of its reactions.
- *Empathy-related responses:* For this study, NAO was designed to adapt to the cognitive level of PwD, for instance by adjusting the difficulty of exercises to each person's capacities, and by being supportive when the participant experienced some difficulties. For some participants NAO laughter facilitated the interaction with it. Fasola and Matarić (2010) have suggested that the motivation to interact with a social robot grows stronger if the interaction is adapted to the user's cognitive capacities. Being empathic, reassuring, and providing the participant with positive feedback (Vallerand, 1983) was in this perspective, another factor that could have added to the acceptance of the robot.

Several studies have highlighted as well the capacity humans have of empathic responses with artificial companions. Suzuki et al. (2015) demonstrated that humans can sympathize with the pain of a robot from a physiological point of view: in a painful situation for a robot, a neuronal response involved in empathic behavior was observed in a group of persons using an EEG (electroencephalogram) measure. Rosenthal-Von Der Pütten et al. (2014) showed an activation of the same emotional neuronal circuits when participants watched some videos showing either a human hurting another human or a human hurting a dinosaur-like robot. Activation was nevertheless more important in situations where humans were harming another human.

In order to better understand the quality of the interactions of PwD with NAO in our study, we used the model of empathy applied to HRI, proposed by Tisseron et al. (2015). This model is structured into four dimensions: (a) the *self-empathy*, empathic relationship with oneself; (b) the *direct empathy*, allowing the attribution of emotions and views to others; (c) the *reciprocal empathy*, thinking that another is able to feel our own emotions; and (d) the *intersubjective empathy*, thinking that others can bring us knowledge about ourselves and our emotional states. In our study eight participants showed direct empathy with the robot, that is, they attributed the robot emotional states and its own perspectives. Two persons showed reciprocal empathy, imagining that the robot was able to guess their emotions, or that the robot had emotions in their regard. One participant, showed intersubjective empathy by telling NAO that his compliments made him proud.

We observed conversely that when empathy-related behaviors toward the robot were absent, or uncommon, the adherence to the RAT appeared to be lower. In our study, the only participant who did not address the robot directly, did not attribute emotions to it, neither used qualifying adjectives when talking to/about the robot, appeared disengaged from the therapeutic activity. In sum, empathy toward the robot seems to be associated to engagement in RAT, but more research is needed to better measure and understand this association.

(e) *The therapeutic framework:* In our study, the therapist was a vehicle for constructive engagement in the CT sessions. The NAO robot, by its social characteristics, its humanoid aspect, and its social and affective behavior, also had the effect of engaging actively PwD. However, it is not possible to conclude that engagement observed in RAT is entirely due to the NAO itself. We observed that the therapist had an essential role in facilitating HRI as well. Indeed, at several times the therapist showed the participant how to talk to the robot or to touch it. The therapist in our framework created a true collaborative relationship with the robot as her assistant, contributing probably to help the participant accept and collaborate with the robot in a similar way. Further studies should explore this finding by comparing engagement of PwD in the three conditions: the therapist alone, the robot alone, and the therapist and the robot working together.

### Studying engagement in RAT

Overall results of this pilot study showed elevated levels of constructive engagement in both conditions (CT and RAT) comparatively higher in the first one. Conversely, passive engagement was more pronounced in RAT sessions. Though these results did not reach statistical significance, they are consistent with Cohen-Mansfield et al. (2010) study in which engagement toward 23 different stimuli, representing different levels of social attributes, was examined in 193 PwD. Results from their study showed higher levels of engagement and more positive attitude toward social, realistic and animated stimuli. Human and live stimuli appeared to be more engaging than non-human and non-alive stimuli. In our study the therapist was a vehicle for constructive engagement in the session. The robot NAO, encompassing most of the previously cited stimuli features that usually engage PwD, incited high levels of constructive engagement as well, even if it was a lower level than a real human (therapist).

From a methodological perspective, we found video-analysis to be a suitable method to examine and measure behavioral and emotional engagement in PwD during the course of an activity. However, the categories of engagement originally used in the MPES (Judge et al., 2000) resulted somehow too general in the context of RAT because they do not allow the distinction between the specific effect of the robot from the effect of the therapist or that from the environment. Also, in the MPES protocol it is not possible to differentiate the specific kind of behavior supporting engagement (e.g., visual, verbal, physical or emotional). This level of detail seems important in order to appreciate the analyze the contribution of robotic mediation. The Video Coding—Incorporating Observed Emotion (VC-IOE) tool developed by Jones et al. (2015) might provide a more coherent and comprehensive method for the assessment of engagement and merits to be tested in future studies.

### Limitations of the study

The present study presents some methodological limitations that should be taken into consideration when interpreting the above presented findings.

First, because of its exploratory nature it included a very limited number of participants and of therapy sessions. Further studies in this area should involve a larger number of subjects and a greater number of sessions in order to investigate RAT effects in the medium and long-term. Also, the sample group in this study was very heterogeneous regarding their clinical profile, aspect that limited the possibility of identifying profiles of respondents. This aspect would be an interesting dimension to examine in future work.

A third limitation refers to the absence of a valid control group. In our pilot study each patient participated only in one CT session but in three RAT sessions. This study design was chosen because of time constraints, with the idea of giving the priority to the observation of RAT sessions while keeping at the same time a baseline evaluation using a conventional therapeutic setting (patient-therapist). Since the assessment of clinical effects of the intervention was not the objective of the research, we accepted to keep the disparity between the two conditions; however, this choice impacted the quality of the results and limited the possibilities of analysis. Further studies should include a control condition truly comparable with the experimental one in terms of contents and frequency.

Finally, the results of this research should also be interpreted taking into consideration the technical possibilities of social robots today. In our experiment the robot NAO was completely controlled by an external operator who used the WOZ technique. Consequently, the observed interactions between NAO and the patients who took part in the study do not reflect to the current capabilities of such a robot. Indeed, we observed very positive HRI during RAT sessions. However, most of these interactions took place between humans: the patient, the therapist and the “wizard” who operated the robot. The fact that the robot behaved very “humanly” could explain why levels of engagement were very similar in the CT condition and in RAT sessions. We believe that this kind of “controlled” experiments are necessary to progress in the definition of the framework of RAT. Nevertheless, it seems important that future studies integrate progressively robot automation in order to examine the real possibilities of HRI with persons with cognitive impairment.

## Conclusion

The results of this exploratory study confirmed the feasibility of robot-assisted psychomotor therapy for PwD. We were able to identify some encouraging indicators in favor of using the NAO robot in such kind of therapeutic program: a very good appreciation of the robot within this context, high positive emotional responses in RAT sessions, a better appreciation of RAT sessions, and a positive correlation between engagement of PwD in RAT sessions and the level of neuropsychiatric symptoms. Indeed, the robot NAO can be considered as a mediating tool favoring patients' engagement in psychomotor therapy when the therapist finds it difficult to motivate and involve the person in the intervention.

After improvement and simplification of the control software a larger trial would help to examine the clinical benefits of this kind of intervention, and to better understand the emotional impact of social robots in PwD. Future studies should also focus on the conception and assessment of other kinds of RAT for dementia care, such as physiotherapy or speech therapy.

## Author contributions

NR and LR have equally contributed to the development of the study, data acquisition, analysis and interpretation. MP conceived and supervised the study. MP, NR, and LR drafted the article, AR, CM, and HL participated in revising it critically. All authors read and gave final approval of the version submitted.

### Conflict of interest statement

The authors declare that the research was conducted in the absence of any commercial or financial relationships that could be construed as a potential conflict of interest.
