# Supplementary material for: Affective and Engagement Issues in the Conception and Assessment of a Robot-Assisted Psychomotor Therapy for Persons with Dementia
Source: Front Psychol. 2017 Jun 30;8:950. doi: 10.3389/fpsyg.2017.00950 (PMC5491651; doi:10.3389/fpsyg.2017.00950)
Supplement: Supplementary file 1 [file Presentation1.pdf]

Supplementary Material

Affective and engagement issues in the conception and assessment of a robot-assisted psychomotor therapy for persons with dementia

Natacha ROUAIX, Laure RETRU-CHAVASTEL, Anne-Sophie RIGAUD, Clotilde MONNET, Hermine LENOIR, Maribel PINO*

* Corresponding Author: maribel.pino@aphp.fr

1 Supplementary Figures

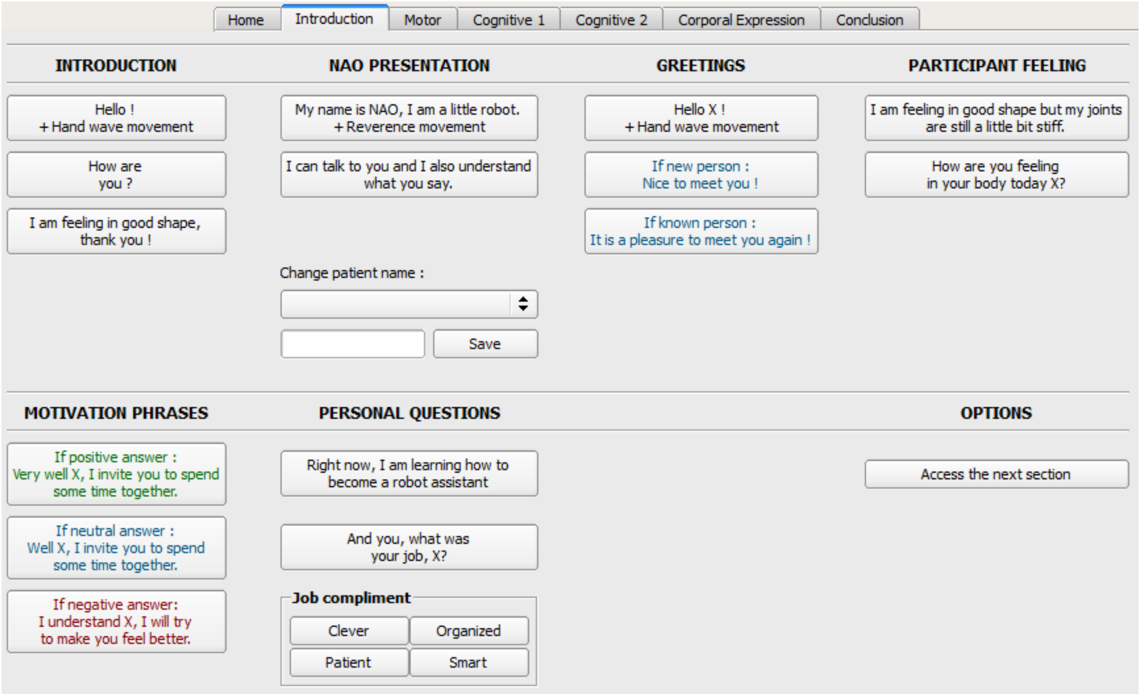

Supplementary Figure 1. Control interface "Introduction section".

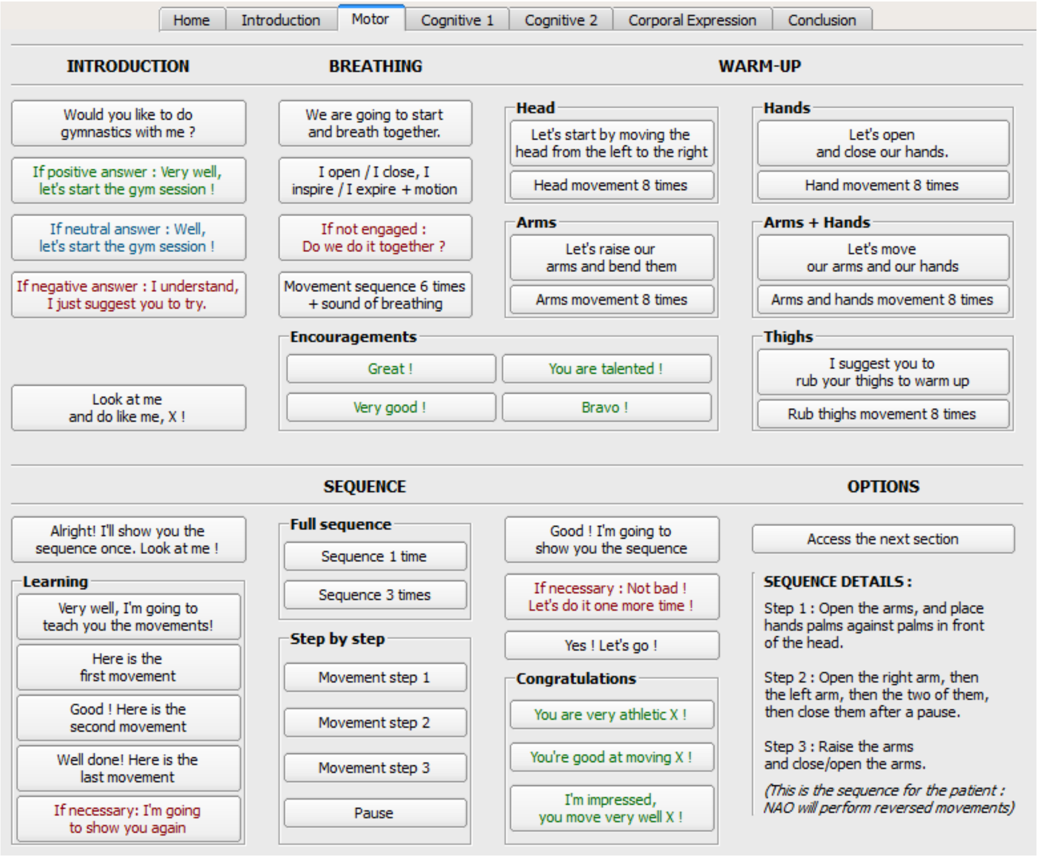

Supplementary Figure 2. Control interface “Motor section”.

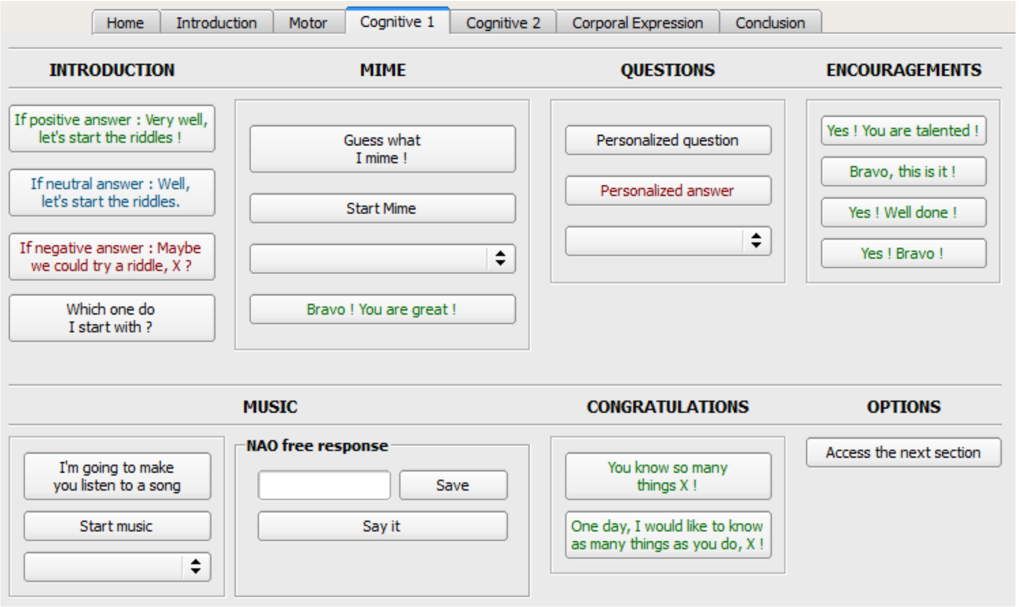

Supplementary Figure 3. Control interface “Cognitive stimulation section 1”.

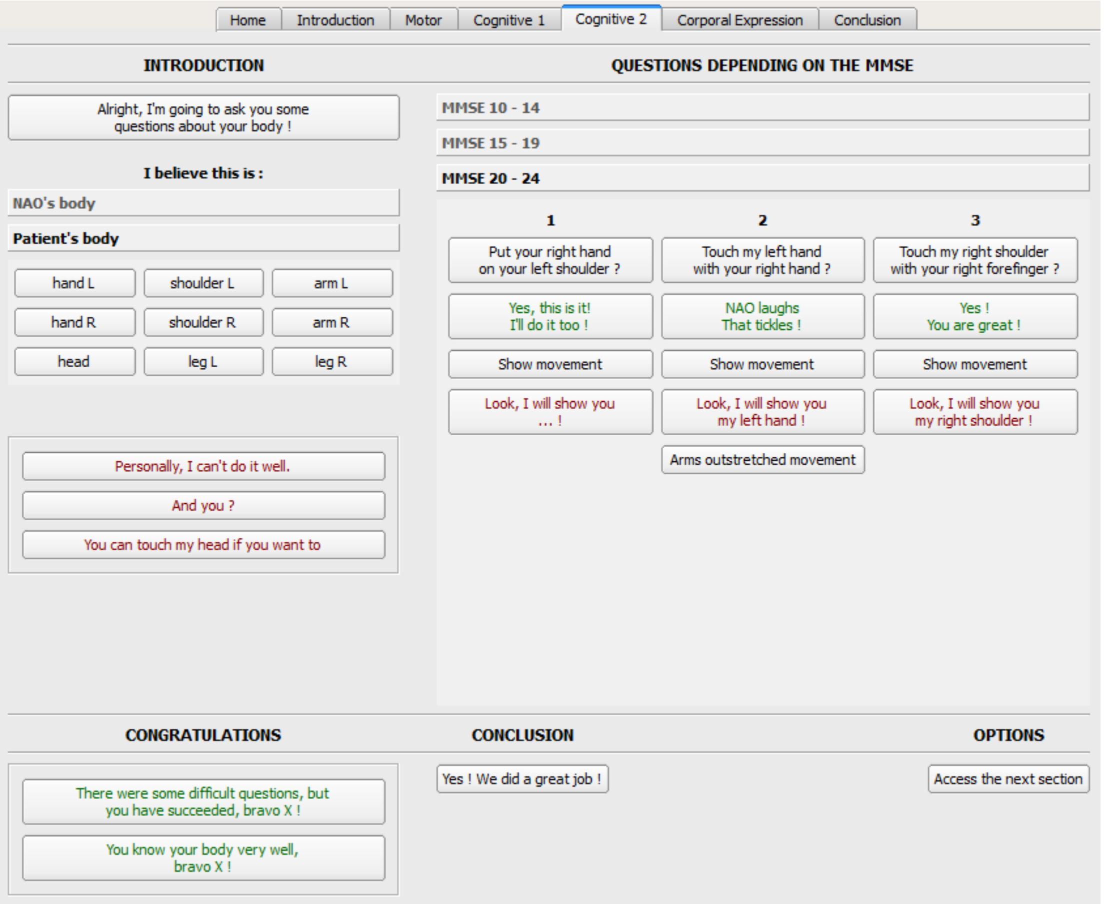

Supplementary Figure 4. Control interface “Cognitive stimulation section 2”.

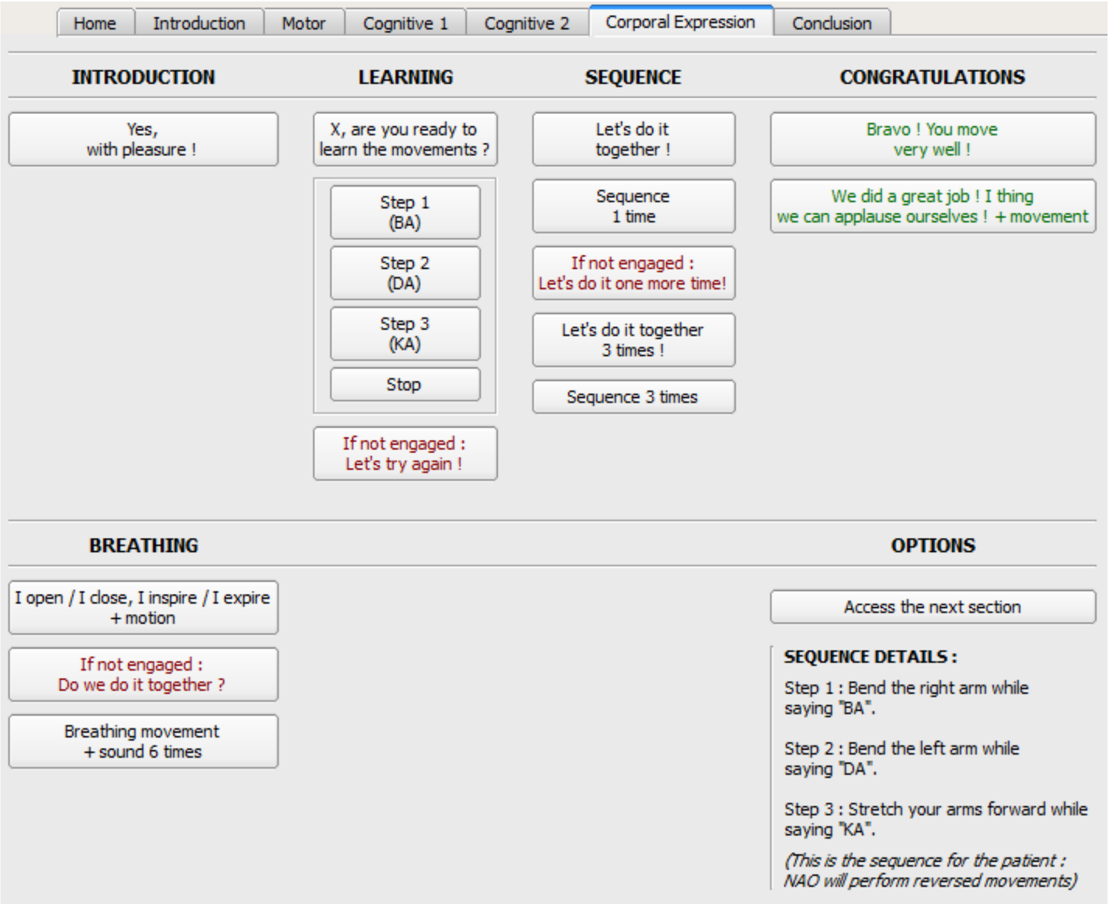

Supplementary Figure 5. Control interface “Corporal expression”.

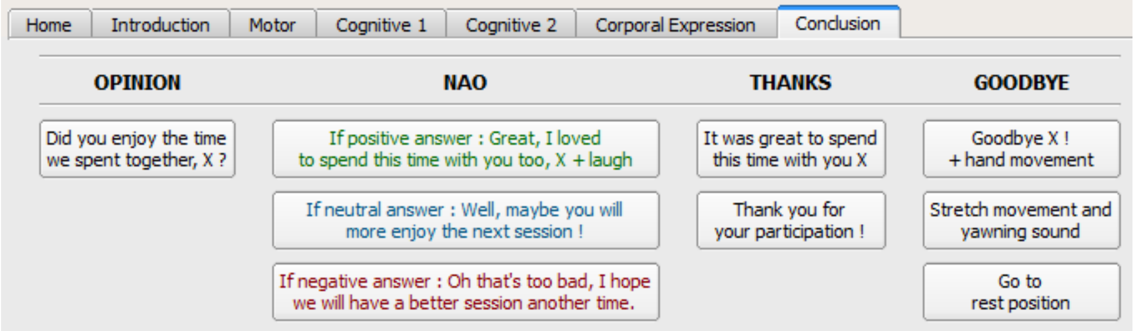

Supplementary Figure 6. Control interface “Conclusion section”.
